# Reversal of Long-Term Weight Regain After Roux-en-Y Gastric Bypass Using Liraglutide or Surgical Revision. A Prospective Study

**DOI:** 10.1007/s11695-020-04856-y

**Published:** 2020-07-21

**Authors:** Fritz F. Horber, Rudolf Steffen

**Affiliations:** 1 Aerztezentrum Reichenburg AG, Kantonsstrasse 60, 8864 Reichenburg, CH Switzerland; 2 Department of Surgery, Klinik Beau Site, Berne, Switzerland

**Keywords:** Bariatric surgery, Roux-en-Y gastric bypass, Liraglutide, Weight regain, Endosurgery, Revisional surgery

## Abstract

**Purpose:**

This study investigates whether pharmacotherapy with liraglutide is similarly effective in reversing weight regain more than 6 years after Roux-en-Y gastric bypass (RYGB) as revisional surgery aimed at restoring restriction.

**Methods:**

Ninety-five consecutive patients (11 male, 84 female; mean BMI 45 ± 6 kg/m^2^) undergoing RYGB 9 ± 4 years ago were treated for 24 months as follows: Patients, who gained less than 10% from weight NADIR, served as controls and were provided lifestyle counseling (DC, *n* = 30). The others were allowed to choose between three different treatment groups: daily s.c. administration of liraglutide (LG, *n* = 34); endosurgery using Apollo’s Overstitch System™ (ES, *n* = 15), or implantation of a Fobi-ring with pouch resizing (FP, *n* = 16).

**Results:**

Controls kept their weight stable during 24 months of study (− 0.1 ± 1.7 kg/m^2^). Weight loss was 4.8 ± 2.9 kg/m^2^ for LG and 5.5 ± 2.9 kg/m^2^ for FP, both losing more than 85% of regained weight from weight NADIR (*p* < 0.001). In contrast, weight loss in ES was 1.0 ± 0.9 kg/m^2^ (i.e., 20% of regained weight). Thirty-seven percent of FP experienced serious complications (*p* < 0.05) in contrast to the other groups. An improved prevalence of hypertension and dyslipidemia was observed in LG and FP (*p* < 0.02) 24 months after intervention.

**Conclusions:**

Weight regain during more than 6 years after RYGB can be safely and effectively reversed with liraglutide. Compared with revisional surgery, pharmacotherapy with liraglutide was low risk and resulted in an important improvement in hypertension and dyslipidemia. Therefore, daily subcutaneous injections of liraglutide are a valid option to treat weight regain after RYGB.

## Introduction

Bariatric surgery (BS) remains the most effective treatment for morbid obesity, offering sustained long-term weight reduction with improvement and prevention of comorbidities and reduced mortality rates [1, 2]. However, up to 25% of patients struggle with considerable weight regain after BS [2–4]. In a NEJM article from 2017 [3] with a patient follow-up of 12 years after Roux-en-Y gastric bypass (RYGB), median weight regain was 10 kg out of a mean maximal weight loss of 45 kg (i.e., a mean regain of weight after weight NADIR of 22.5%). An Indian study [4] of 9617 patients followed up over 5 years after RYGB demonstrated a significant weight regain of more than 25% after weight NADIR in 14% of patients (i.e., 1336 of initial 9617 patients). It is well known that weight regain is associated with reappearance or worsening of obesity-associated comorbidities like hypertension and type 2 diabetes (T2D): 75% experience remission after 2 years and 51% after 12 years [5]. Therefore, it is important to address weight regain after RYGB surgery, to avoid compromising the sustained improvement of obesity-associated comorbidities [3] and to improve long-term patient satisfaction.

Reoperations are associated with serious complications in all surgery. Bariatric surgery is especially prone to late revisions due to insufficient weight loss or weight regain, which results in increased complications and higher mortality rates [6–11]. Endosurgery for dilated upper anastomosis and weight regain was evaluated in a 2018 meta-analysis [12] and considered a valid alternative to laparoscopic surgical reoperation for weight regain, as a much lower complication rate was recorded [12] compared with laparoscopic reoperations [6–11].

Pharmacotherapy with liraglutide, a glucagon-like peptide1 (GLP1) receptor agonist, has been successful in reducing weight in non-surgical patients with obesity in its 3.0 mg daily subcutaneous injection and was reported to result in a mean weight loss of about 9 kg after 1 year in non-surgical patients [13]. Liraglutide, therefore, presents an interesting alternative to reoperation or endosurgery in the management of weight regain after BS. In a preliminary study of short duration (28 weeks) [14] in RYGB patients, adjunctive high-dose liraglutide resulted in a median weight loss of 4.7 kg/m^2^. Similarly, a study in RYGB or gastric banding patients [15] resulted in a median weight loss of 7 kg after 8 months. Moreover, in a short-term randomized clinical trial of 28 weeks, patients with obesity were successfully and safely treated with subcutaneous liraglutide (1.8 mg daily) for persistent or recurrent T2D [16] more than 1 year after bariatric surgery (RYGB or sleeve resection). Studies comparing medical, surgical (i.e., reoperation or endosurgery), or lifestyle interventions for managing insufficient weight loss or weight regain more than 6 years after RYGB surgery are scarce [17] and need further medical validation.

Since more than 80% of weight regain has been previously observed within the first 6 years after the operation [3], we included 95 consecutive patients who had undergone a RYGB at least 6 years previously presenting for yearly controls in the outpatient practice. These patients were assigned to different groups: initiation of liraglutide^.^ [5, 13–15], endosurgery of the upper anastomosis [12], or laparoscopic implantation of a Fobi-ring with pouch resizing [11, 17]. Patients who kept their weight through lifestyle treatment alone [18] served as control.

With the study, we aim to further elucidate whether pharmacotherapy with liraglutide is similarly effective in reversing weight regain more than 6 years after RYGB as revisional surgery aimed at restoring restriction.

## Materials and Methods

### Patients

Between January 2016 and March 2018, 95 consecutive unrelated Caucasian Swiss patients with formerly severe obesity, having been operated for RYGB at least 6 years earlier, were included in the study, independent of reported weight regain (Table 1). Inclusion criteria were as follows: RYGB at least 6 years ago, age older than 18 years, and regular follow-up at least every 6 months at the outpatient consultation of the obesity specialist (FH) after their primary RYGB operation. All patients were covered by public health insurance. Health insurance of individual patients was not a factor in deciding which treatment patients chose. Exclusion criteria were as follows: patients had received bariatric procedures other than RYGB or patients with a history of psychosis or alcohol or drug abuse.

**Table 1:** Baseline patient characteristics before RYGB and at the beginning of treatment of weight regain

Group	N	Sex^+^	Age^+^	Weight^+^	BMI^+^	BMI at NADIR*	WRG^&^	BMI at start of study	Years
		m/f^#^	years	kg	kg/m^2^	kg/m^2^	%	kg/m^2^	postop^$^
Total	95	11/84	54 ± 12	122 ± 18	45 ± 6	25.7 ± 3.8	18 (0–52)	30.4 ± 5.1	9 ± 4
DC (controls)	30	6/24	55 ± 10	124 ± 20	44 ± 9	25.5 ± 3.4	6 (0–9.7)	27.1 ± 5.0	9 ± 4
LG (liraglutide)	34	3/31	56 ± 10	120 ± 19	45 ± 8	25.5 ± 4.4	25 (11–46)	31.2 ± 4.0	9 ± 5
ES (endosurgery)	15	0/15	55 ± 12	115 ± 10	43 ± 4	24.9 ± 3.8	20 (11–52)	31.0 ± 4.2	8 ± 4
FP (Fobi)^§^	16	3/13	43 ± 11^£^	132 ± 16^£^	46 ± 5	27.5 ± 3.3	27 (13–43)	34.2 ± 4.9	8 ± 3

Values are given as mean ± standard deviation (SD)

^+^Sex, age, weight, and BMI (body mass index) are given at the time of RYGB surgery

*NADIR: lowest weight reached 19 ± 7 months after implantation of RYGB

^&^WRG: maximum weight regained after weight NADIR before entering the study (9 ± 4 years after the primary operation) expressed in % increase from weight NADIR (100%/BMI at NADIR); * BMI at beginning of study

^#^m: male; f: female

^$^postop: years after implantation of RYGB

^§^Laparoscopic pouch revision with Fobi-ring [11, 17]

^£^*p* < 0.01 vs all other groups (analysis of variance, ANOVA)

At the regular outpatient half-yearly bariatric control visit, at least 6 years after RYGB, patients were divided into 4 groups. Patients who had gained less than 10% from their respective measured weight NADIR (median 6% (range 0–9.7%), Table 1) were enrolled as controls. They were provided with dietary and lifestyle counseling at least every 3 months up to 24 months after study initiation (group DC, *n* = 30) to address their eating behavior, physical activity, substance use, and weight control practice, similarly as suggested by King et al. [18]. All patients who suffered from considerable weight regain (> 10% from weight NADIR) were offered one of the following three therapeutic possibilities in addition to dietary and lifestyle counseling [18]:

- Treatment with subcutaneous self-injection of the Glucagon-like peptide 1 receptor (GLP-1) agonist liraglutide (Novo Nordisk Pharma AG, Zurich, Switzerland), to a maximal dose of 3.0 mg daily, if tolerated, to enforce satiety (group LG, *n* = 34) [13]. Median weight regain from weight NADIR was 25% (range 11–46; Table 1).
- Endosurgery, using Apollo’s Overstitch™ suturing system to reinstall restriction (group ES, *n* = 15) [12]. Median weight regain from weight NADIR was 20% (range 11–52; Table 1). For operative details, see below.
- Surgical resizing of the gastric bypass pouch with additional implanting of a Fobi-ring to reinstall restriction (group FP, *n* = 16) [6, 11, 17]. Median weight regain from weight NADIR was 27% (range 13–43; Table 1). For operative details, see below.

Weight regain of 10% was chosen as a cut off for offering one of the three treatment modalities. The rationale for that was that internal evaluations of the quality of life questionnaires 8 years after BS revealed that weight regain of more than 10% was reported to have the most important negative impact on patients’ quality of life (unpublished data FH).

All patients were followed by the same obesity specialist (SCOPE Fellow, FH) at his private practice, Ärztezentrum Reichenburg, Switzerland. All weights reported in text and tables were measured at the outpatient consultation of the obesity specialist (FH).

Patients were fully informed and gave written consent to the study protocol and their participation in the study. All procedures performed were in accordance with ethical standards of the national research committee. The study complied with the Declaration of Helsinki 1964 and its later amendments.

### Operations

#### Laparoscopic Roux-en-Y Gastric Bypass

RYGB creates a stapled small proximal gastric reservoir attached to the jejunum, bypassing the stomach, pylorus, duodenum, and first part of the jejunum as previously described [9, 10].

#### Laparoscopic Pouch Resizing with Fobi-Ring [11, 17]

After the placement of trocars and dissecting the liver off the bypass, the anatomical landmarks of the pouch are dissected. The pouch is completely freed circular under careful sparing of nutritional blood supply to the lesser curvature. The anesthesiologist insufflates air through a gastric tube while the jejunum under the anastomosis is clamped. To reduce the dilated pouch—most often laterodorsal—a 35 French bougie is inserted trans-orally and the pouch is downsized along with the bougie using linear staplers or occasionally in case of only minimal distension using plicating stitches. After downsizing of the pouch, a Fobi-ring (Mini-Mizer Fobi-ring by Bariatric Solutions GmbH, Stein am Rhein, Switzerland) is placed 0.5–1 cm above the anastomosis in a perigastric technique thus avoiding the necessity of additional sutures. The length of the Fobi-ring can vary between 5.5 and 6.5 cm depending on the scar tissue present. Post-operative management included counseling by a dietician regarding the recommended 2 weeks of liquid diet, proton pump inhibitors 40 mg twice a day, orally, for at least 4 weeks, and pain medication as necessary.

#### Endosurgery [12]

All procedures were performed by the same team (surgeon JZ and gastroenterologist HM) at the same bariatric surgery center. All procedures were performed under general pharynx, and proximal esophagus, the gastrojejunal (G-J) anastomosis, was primed circumferentially with an argon laser. The OverStitch™ Suturing Device (*Apollo* Endosurgery, SurgTech AG, Baar, Switzerland) was introduced and the narrowing of the G-J anastomosis was performed using 1–2 sutures, each with 4–6 stitches. A final inspection of the patency of the anastomosis was performed before the removal of the over-tube. The size of the G-J anastomosis, size of the pouch, the duration of operation, the number of sutures, and complications were recorded. Patients were observed overnight in the hospital post-operatively as per hospital policy. Post-operative management included counseling by a dietician regarding the recommended 2 weeks of liquid diet, proton pump inhibitors 40 mg twice a day, orally, for at least 4 weeks, and pain medication as necessary.

### Comorbidities and Their Reporting

Comorbidities listed were reported using the following criteria: Hypertension was defined as blood pressure ≥ 130/85 mmHg or taking antihypertensive drugs. Type 2 diabetes (T2D) was reported when HbA1c ≥ 6.5% or taking antidiabetic drugs; Dyslipidemia was reported with triglyceride levels > 200 mg/dl, HDL-cholesterol < 35 mg/dl, LDL-cholesterol levels > 100 mg/dl, or taking lipid-lowering drugs.

Data at 24-month follow-up were reported as follows: improved, improvement in measured values at 2 years compared with baseline or reducing dosage of respective drug treatment; impaired, impairment in measured values at 2 years compared with values before study start or increasing dosage of respective drug treatment; remission, normalization of measured values and stop of all medication to treat comorbidities.

### Follow-up Schedule

The patients had routine follow-up appointments at the outpatient private practice with the same certified SCOPE Fellow at 4 weeks, 8–12 weeks, 6, 9, 12, 18, and 24 months after the procedure or liraglutide initiation, with more frequent consultations as indicated by patient symptoms. Patients’ weight and vitals were taken at each visit using the same equipment. All post-operative procedures were recorded.

### Statistics

Mean ± SD or median (range), where appropriate, is presented throughout the manuscript unless specified otherwise. Differences between groups were calculated using analysis of variance (ANOVA), ANOVA for repeated measures, Chi-squared test, Kruskal-Wallis, or Friedman test, where appropriate. All analyses were performed using SAS (version 9.3) or IBM SPSS (version 22).

## Results

### Baseline Characteristics and Long-Term Weight Regain After Bariatric Surgery

Ninety-five consecutive patients with a mean BMI (body mass index) of 45 ± 6 kg/m^2^ before RYGB surgery were included, 88% women vs 12% men (Table 1). The mean time since their primary operation was 9 ± 4 years. Mean BMI at the beginning of the study was 30.4 ± 5.1 kg/m^2^. The median weight regain of the whole group from weight NADIR was 18% (range 0 to 52%) (Table 1). BMI at weight NADIR and the start of the study was slightly higher in patients treated with Fobi-ring and pouch resizing (*p* < 0.05 vs other groups, ANOVA). Twenty-eight percent of patients enrolled were further treated with dietary and lifestyle counseling (DC, *n* = 30) because of their only slight increase in median weight after weight NADIR of 6% (range 0 to 9.7%) (Table 1) and were used as controls. Thirty-four patients chose painless self-injection of liraglutide (up to 3.0 mg, daily subcutaneous) to treat weight regain (median 25%, range 10.5–46.2%; Table 1). Fourteen percent of participants chose endosurgery (ES) to treat their median weight regain of about 20% (range 10.5–51.9%; Table 1). Mean observation time after implantation of RYGB of the ES group was slightly but not significantly shorter than the LG or DC group (Table 1). Fourteen percent of patients decided to have Fobi-ring implantation with pouch resizing (FP) in response to a median weight regain of 27% (range 12.5–42.8%; Table 1). Subjects treated with pouch resizing and Fobi-ring implantation were significantly younger and heavier than all other groups at the time of reoperation (*p* < 0.05, ANOVA). Prevalence of hypertension, T2D, and dyslipidemia was not statistically different between the groups investigated before initiation of treatment (Kruskal-Wallis test).

### Impact of Treatment Modality on Weight Regain after RYGB

#### Group DC - Control Subjects (Table 2)

During the 24 months of observation (approximately 10–11 years after primary surgery), mean weight remained stable at 6% above weight NADIR (delta BMI − 0.1 ± 1.7 kg/m^2^, Table 2) in the control group (DC), with only dietary and lifestyle counseling at least every 3 months [18]. No patient desired to change to a treatment group throughout the 24 months of observation. No treatment-associated side effects were encountered. Follow-up after 24 months was 100%. Before the initiation of lifestyle intervention, prevalence of drug-treated hypertension was 33% (10 out of 30 patients), a rate that did not change during 2 years of observation. One patient had drug-controlled T2D throughout the study period with stable HbA1c, whereas another patient developed prediabetes with an HbA1c of 6.1% at the end of the study. Dyslipidemia was detected in 53% of patients before lifestyle intervention and remained similar during the 24 months of observation.

**Table 2:** Impact of treatment modality on weight regain 9 years after RYGB

Group	N	BMI-0^+^	BMI-24*	delta BMI-lost	Follow-up of weight change (kg) after intervention (months)						
		kg/m^2^	kg/m^2^	kg/m^2^	0 months	3 months	6 months	9 months	12 months	18 months	24 months
DC (controls)	30	27.1 ± 5.0	27.2 ± 4.5	− 0.1 ± 1.7	75 ± 15	75 ± 15	75 ± 15	76 ± 14	76 ± 13	75 ± 13	75 ± 13
LG (liraglutide)	34	31.2 ± 4.0^#^	26.4 ± 3.5	4.8 ± 2.9^£^	84 ± 13^#^	80 ± 13	77 ± 12	76 ± 12	74 ± 11	73 ± 10	72 ± 9^£^
ES (endosurgery)	15	31.0 ± 4.2^#^	30.0 ± 4.4^$^	1.0 ± 0.9	83 ± 14^#^	80 ± 14	80 ± 14	80 ± 14	80 ± 14^$^	---------	---------
FP (Fobi)^§^	16	34.2 ± 4.9^#^	28.7 ± 4.6	5.5 ± 2.9^£^	96 ± 12^#^	90 ± 12	88 ± 12	85 ± 12	83 ± 11	82 ± 12	79 ± 10^£^

Values are given as mean ± standard deviation (SD)

^+^BMI-0 depicts BMI at the beginning of liraglutide therapy, endosurgery, or Fobi-ring implantation, respectively

^*^BMI-24 depicts BMI after 24 months of liraglutide therapy or after Fobi-ring implantation and 12 months after endosurgery, respectively

^&^Daily dose of subcutaneous liraglutide at 24 months (m): 2.0 ± 0.9 mg (range 0.6 to 3.0 mg)

^#^*p* < 0.01 controls vs other groups before treatment (ANOVA)

^$^All patients demanded additional drug therapy after 12 months of treatment

^§^Laparoscopic pouch revision with Fobi-ring [11, 17]

^£^*p* < 0.001¸ ANOVA for repeated measures 0 vs 24 months of treatment

#### Group LG - Subjects Treated with Liraglutide for 24 months After RYGB (Table 2)

During the 24 months of observation, mean weight loss was 13 ± 8 kg corresponding to a reduction of BMI of 4.8 ± 2.9 kg/m^2^ (*p* < 0.001 before vs 24 months of treatment, ANOVA for repeated measures; Table 2) in patients treated with liraglutide with a median daily tolerated dose of 2.0 mg (range 0.6–3.0 mg). The reduction of BMI throughout the 24 months of study was higher than 1 kg/m^2^ in 31 patients (responders, 91%). Only three patients (9%) were declared non-responders, losing less than 1 kg/m^2^ BMI or less than 5% of weight during the 24 months of liraglutide treatment [13]. During years 10 to 11 after the primary RYBG surgery, the secondary weight gain of the whole group was reduced by a mean of 87% (*p* < 0.001, ANOVA for repeated measures). As a consequence, the mean weight compared with weight NADIR was only missed by 3% (not significant (NS) vs NADIR). Side effects of liraglutide were scarce except for nausea. Nausea in the initial weeks of drug therapy was quite common necessitating a slower increase in daily dosage and resulting in 50% of patients not reaching a maximal dose of 3.0 mg as recommended by the manufacturer due to persistence of gastrointestinal side effects. The dose could be increased to a median dose at 24 months of 2.0 mg (range 0.6–3.0 mg). No correlation between the maximum tolerated daily dose of liraglutide and weight loss could be detected. Also, other evident predictive factors for non-response could not be observed. Follow-up after 24 months was 100%; thus, no patient had to discontinue liraglutide treatment. Interestingly, no patients reported gastroesophageal reflux disease during the study period. Before initiation of liraglutide, the prevalence of drug-treated hypertension was 29% (10 out of 34 patients). After 2 years of liraglutide therapy, antihypertensive drug treatment could be reduced in 7 (70%) and stopped in 3 (30%) patients with measured values of < 130/85 mmHg (*p* < 0.01, Friedman test). Two patients had T2D at the beginning of the study. Both normalized their HbA1c during the 2 years of follow-up with continuing liraglutide treatment. Prevalence of dyslipidemia was 50% (17 out of 34 patients) before the initiation of liraglutide therapy. After 2 years of liraglutide-induced weight reduction, dyslipidemia improved in 62% (*n* = 10) of patients (*p* < 0.01, Friedman test).

#### Group ES - Subjects Treated with Endosurgery [12] for 12 months After RYGB (Table 2)

During the 12 months of observation, mean weight loss was 3 ± 3 kg (delta BMI 1.0 ± 0.9) in patients with endosurgery (NS before vs 12 months after treatment, ANOVA for repeated measures). During year eight after primary operation, weight regain was reduced by only 20% (NS before vs 12 months after endosurgery, ANOVA for repeated measures) in subjects treated with endosurgery despite narrowing the stoma diameter of the upper anastomosis of RYGB from 22 ± 3 to 6 ± 2 mm (*p* < 0.001). Because of dissatisfaction with their weight loss, all patients wanted another solution to treat weight regain since their mean weight after 12 months was still 12 kg or 5.1 ± 3.9 kg/m^2^ above their respective weight NADIR (*p* < 0.01 vs NADIR). One patient needed two gastroscopies because of unexplained upper abdominal pain and reflux without further intervention. All patients returned on a regular base for follow-up during the 12 months. Before the endosurgery, prevalence of drug-treated hypertension was 27% (4 out of 15 patients), a rate that did not change during the observational period. No T2D was detected during the study interval. Dyslipidemia was recorded in 53% of patients before endosurgery and remained at the same rate throughout.

#### Group FP - Subjects Treated with Laparoscopic Pouch Resizing with Fobi-Ring [11, 17] (Table 2)

During the 24 months of observation, mean weight loss was 17 ± 7 kg in patients with pouch resizing and Fobi-ring (*p* < 0.001 before vs 24 months after treatment, ANOVA for repeated measures). During years 9 to 10 after the primary RYBG surgery, secondary weight gain was reduced by more than 85% (*p* < 0.001, ANOVA for repeated measures) 24 months after revisional surgery. As a consequence, the FP patients’ mean weight was only missed by 4% to weight NADIR (NS vs NADIR). More than a third of the group (*n* = 6) experienced complications after revisional surgery: One patient suffered severe pain in the upper abdomen 6 days after surgery necessitating hospitalization. No further intervention was necessary and pains resolved within 3 days. Another patient experienced an internal hernia within the first year after intervention necessitating laparotomy. In one patient, the penetration of Fobi-ring within the second year after revisional surgery had to be resolved by endoscopic removal. Three patients needed gastroscopy with a total of 37 endoscopic dilatations (patient 1, 32 times; patient 2, three times; patient 3, two times with concomitant removal of a meat bolus). No mortality or leak-associated sepsis events were observed.

Patients in group DC, LG, or ES reported only minor complications throughout the study, while 37% of patients with laparoscopic pouch resizing with Fobi-ring implantation (FP group) experienced serious complications (*p* < 0.05 vs all other groups, Chi-squared test) during the 24 months after revisional surgery. Follow-up after 24 months was 100%. The prevalence of drug-treated hypertension was 19% (3 out of 16 patients) before revisional surgery. Two years later, antihypertensive drug treatment could be reduced in two and stopped in one patient. No T2D occurred during the 24-month observation period. Dyslipidemia was detected in 50% of patients before the revisional intervention; 2 years later, dyslipidemia improved in more than 2/3 of affected patients (*p* < 0.01, Friedman test).

## Discussion

Long-term weight regain after bariatric surgery (i.e., after more than 6 years) is a frequent, bothersome clinical problem for patients [2, 3]. The present study demonstrates that liraglutide administered subcutaneously at a daily dose up to 3.0 mg when used as an adjunct to lifestyle counseling results in similar mean weight loss as in non-operated patients treated with liraglutide in a previous study [13] with minimal side effects. Interestingly, no patient-reported gastroesophageal reflux disease occurred during the 24 months of observation. Moreover, patients with liraglutide treatment lost 15 ± 5% of their body weight (i.e., 13 ± 4 kg) after 24 months, achieving a similar weight to their weight NADIR after RYGB (Table 2). This finding is in line with a recent short-term study of 20 patients with a loss of mean weight of 9.7% in 28 weeks [14] or 7 kg after 8 months [15]. It is of interest to note that in the present study, the mean change in body weight was also stable after 24 months of treatment as long as patients continued liraglutide treatment (weight at 30 months (*n* = 23); 74 ± 10 kg and 36 months (*n* = 18) 73 ± 10 kg).

In a 2017 retrospective analysis, Stanford et al. [19] demonstrated that pharmacotherapy-induced weight loss might be a useful adjunct in patients with inadequate weight loss or weight regain after bariatric surgery. In that study, 54% of patients lost 5% of their total weight with concomitant medication after surgery; 30% lost more than 10% of their weight, and 15% more than 15% [19]. In our study population, using liraglutide resulted in a mean weight loss of 15 ± 8% during the 24 months of treatment: 50% of patients lost more than 15%, 76% lost more than 10%, and 89% lost more than 5%. Interestingly, the percentage of RYGB patients in the present study losing less than 5% of the initial weight was only 11%, whereas in liraglutide, treated non-bariatric patients with a similar initial BMI, more than 35%, did not lose 5% of initial weight [13]. Therefore, liraglutide seems to be the most efficient drug to date to treat inadequate weight loss or weight regain after bariatric operations [19]. Moreover, liraglutide appears to be at least as effective as weight loss adjunctive in bariatric patients as compared with similarly treated non-bariatric patients with obesity [13]). Whether earlier treatment of weight regain (i.e., before 9 years after RYGB) would result in similar weight loss needs further investigation. However, from a patient’s perspective, we would speculate that an earlier intervention might lead to better long-term preservation of quality of life.

It is of interest to note that liraglutide-treated bariatric patients lost a similar amount of weight (13 ± 8 kg or BMI 4.7 ± 2.9 kg/m^2^) as did surgically revised patients (17 ± 7 kg or BMI 5.5 ± 2.9, NS vs liraglutide, Table 2) during the 24 months of study. This interesting finding raises the question of whether treatment of weight regain after RYGB surgery should primarily be treated with liraglutide, and as a consequence, revisional surgery only be considered for patients not responding to liraglutide treatment. This question is of obvious clinical importance since reoperations after bariatric surgery are associated with a relatively high rate of complications [6–11]. This is also demonstrated in the present study with a rate of 33% complications in surgically revised patients [20]. Moreover, reports on long-term outcomes after surgical gastric pouch revisions are rare with only small patient series [21–23]. Besides the relatively high rate of major complications in these patients, weight loss appears to be inconsistent from zero up to 70% excessive weight loss with a substantial rate of major complications [21–23] pointing to liraglutide as first-line treatment of weight regain after RYGB rather than surgery.

A third of patients in the FP group experienced complications that lead to hospitalization in two cases or endoscopic dilatation of the upper anastomosis (up to 32 times) due to an inability to eat and vomiting. In our study, gastrointestinal disorders were common and mostly transient side effects in all groups that received revisional surgery (group FP and ES). In contrast, liraglutide-treated patients demonstrated a safety profile that is consistent with findings in previous reports for non-operated patients [13, 14, 24, 25]. Other then reported for non-operated patients with obesity [5, 13, 24–26], the liraglutide dose in our study population could not be increased to the maximal daily dose of 3.0 mg in 50% of patients because of the persistence of gastrointestinal side effects. A 2018 study reported that postprandial GLP-1 concentrations increase after RYGB [27]. This finding could potentially explain the need for a smaller dose of liraglutide than that recommended for non-operated patients with obesity.

Gallbladder-related events and pancreatitis were not seen in patients with RYGB and treated with liraglutide in the present study. This is in contrast to non-operated patients, who experience more frequent gallbladder-related events and pancreatitis with liraglutide treatment than with placebo [24–26]. No serious cardiovascular event was observed during the study period in either the liraglutide or in the surgically treated groups. Whether more frequent side effects would occur in larger patient groups treated with liraglutide after bariatric surgery is to be verified in further studies.

As was the case for liraglutide-treated patients, patients treated with endosurgery for weight regain experienced only minor side effects such as reflux disease, which was easily treatable with proton pump inhibitors. However, in contrast to a recent meta-analysis [12] and another study comparing drug treatment with endosurgery [28], in the present study, endosurgery only prohibited further weight regain (Table 2), thus not allowing the patients to significantly lose weight [29]. As a consequence, all patients in the ES group were not satisfied with this treatment modality and stopped the study after 12 months (Table 2). Whether using other or more frequent suturing techniques to reverse weight regain after bariatric surgery as demonstrated by Patel et al. [30] would allow similar results to reverse weight regain after RYGB, as achieved with liraglutide or Fobi-ring implantation with pouch resizing, awaits further study.

As expected and as described before [2, 3] for non-bariatric patients with obesity, liraglutide was able to improve hypertension, T2D, and dyslipidemia impressively as a result of weight loss in RYGB patients treated with liraglutide or Fobi-ring implantation with pouch resizing for weight regain more than 6 years after initial RYGB. In contrast, RYGB patients treated with lifestyle treatment (DC) and patients treated with endosurgery (ES) experienced mostly no change in comorbidities, possibly due to the non-significant weight change during 24 months of study.

There are several limitations to this study, foremost being the selection and ascertainment bias. Additionally, one could criticize the relatively small sample size in each group; however, the fact that the retention rate was 100% in all groups after 2 years strengthens the validity of the results. Furthermore, the study was conducted in only one obesity center. Whether the presented results can be extrapolated to other centers remains open. However, this could be also seen as a positive point that adds to the comparability of our observations, as it excludes all variations in study conduct and analysis. We are aware that randomization to the treatment groups would have been the best approach to increase the scientific validity of our research findings and to answer the proposed question, how to best treat weight regain after bariatric surgery. However, we believe that a randomization approach would have severely decreased the interest to participate in the study, increased the drop-out rate, and most certainly reduced adherence to the treatments. Hence, we would have had less valid data to report and share.

## Conclusion

Up to 3.0 mg of once-daily subcutaneous liraglutide, as an adjunct to lifestyle counseling, was associated with statistically significant weight loss and considerable improvement of comorbidities like hypertension, T2D, or dyslipidemia in patients with more than 20% of weight regain after RYGB as recently suggested by Linke et al. and others [14, 15, 23]. Based on the present results, obtained in one obesity center in Switzerland, we recommend using liraglutide as first-line therapy for the treatment of weight regain after RYGB. Surgical revision should only be considered if patients do not sufficiently respond to liraglutide treatment, a finding not seen in the present study. Whether weight regain observed after other bariatric treatment options such as sleeve resection would respond in a similar way to liraglutide treatment needs to be investigated in detail in future studies.
